# The Role of Dentist in Palliative Care Team

**DOI:** 10.4103/0973-1075.68408

**Published:** 2010

**Authors:** Rani P Mol

**Affiliations:** Department of Oral Medicine and Radiology, Government Dental College, Thiruvananthapuram, India

**Keywords:** Dentistry, Oral cavity, Palliative care

## Abstract

The palliative doctor gives the ‘touch of God’ as he/she takes care of the terminally ill patient. The oncologist encounters great difficulties in managing oral cavity problems of these patients. A trained dental doctor can help other doctors in dealing with these situations. But the general dental surgeon does not have enough idea about his part in these treatments. The community is also unaware of the role that a nearby dentist can play. Adequate training programs have to be conducted and awareness has to be created. A trained dentist will be a good team mate for the oncologist or radiotherapist or other doctors of the palliative care team. In this paper, a brief attempt is made to list a few areas in which a palliative care dentist can help other members of the palliative care team and also the patient in leading a better life.

## INTRODUCTION

The palliative care team consists of specialists in various fields of medicine who provides care and treatment to the patients. A dentist can help to improve the quality of life of the patients. Mouth is the most important organ of expression and it is most often affected in later stages of diseases. Oral cavity is home for a great number of micro organisms which aggravates the disease process. The patients need the help of a dentist to alleviate his discomfort and to live a better life. He can help the patient right from the initial diagnosis of the condition up to the relief of pain in the terminal stages of the diseases. But many a times the general dentist is unaware of his responsibilities toward a terminally ill patient. The community is also unaware of the role that a nearby dentist can play. In this paper, a brief attempt is made to list a few areas in which a palliative care dentist can help other members of the palliative care team and also the patient in leading a better life.

## PALLIATIVE CARE DENTISTRY

Palliative care dentistry was defined by Wiseman[1] as the study and management of patients with active progressive and far advanced disease in whom the oral cavity has been compromised either by the disease directly or by its treatment; the focus of care is quality of life. The dentist may come across the solid tumors of head and neck region or the oral manifestations of hematological malignancies.[2] We are focusing on the squamous cell carcinoma of head and neck region (SCCHN) as they are the most common in the head and neck region.

The steps where a dentist can play his role in the palliative care team are discussed in the subsequent paragraphs.

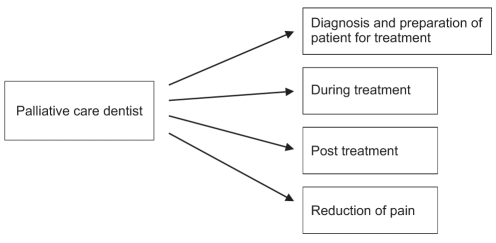



## DIAGNOSIS AND PREPARATION OF PATIENT FOR TREATMENT

**Figure 1:**
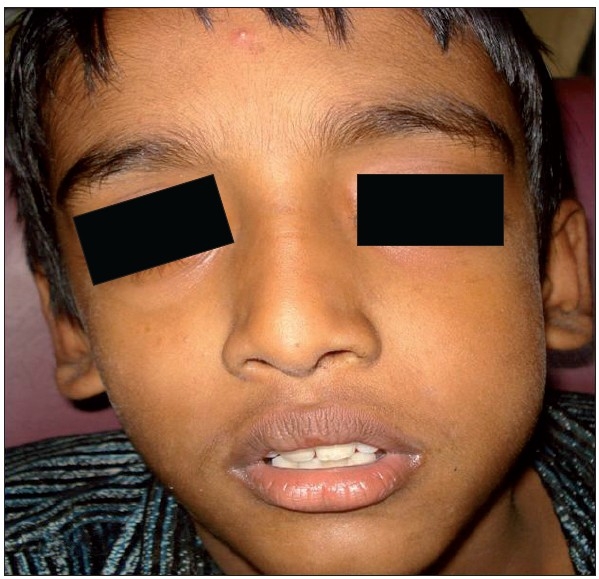
A 12-year-old boy who presented with epiphora

Dentist may be the first person to diagnose the case of head and neck cancer. Owing to neighboring vital structures, SCCHN are complex and require a multidisciplinary approach.[2] The patient can present with a variety of symptoms such as swelling, ulceroproleferative lesions, nonhealing ulcerations, nonhealing extraction socket, difficulty in swallowing, epiphora etc. He should be able to identify and relate the symptoms. Here is a rare cancer case of a 12-year-old boy who presented with epiphora [Figure 1].

A detailed history taking is important giving due care for the patients own words. Give adequate importance to patient’s feelings and spend some time listening to his experiences. Detailed medical and dental history, review of systems, drug allergies, current medications and comprehensive oral examination should be done and recorded for further reference.

**Figure 2:**
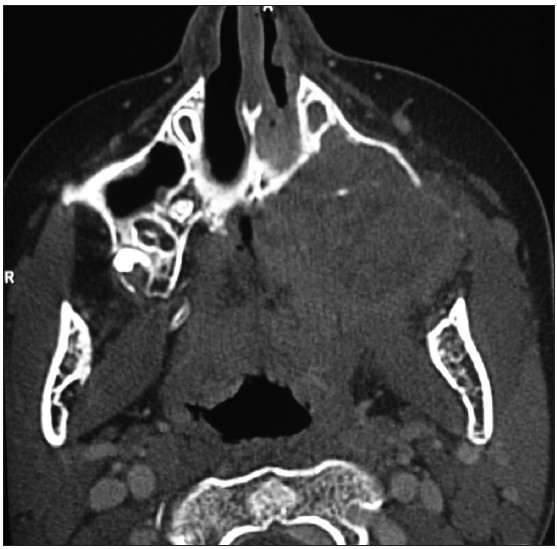
CT scan of the patient in Figure 1 showing extensive destruction of maxillae

Imaging modalities like radiographs, CT scans, MRI and PET scans may be used [Figure 2]. The staging should be done using the standard TNM staging for ease further treatment and follow-ups.[2]

The main role of the doctor is in communicating to the patient and his relatives about the disease. After adequate tests, try to break the ‘bad news’ to the patient and his relatives in kind and affectionate words. Listen to the patients’ response and support his emotional breakdown. Clarify all his doubts and explain to him about the treatment options. While preparing the patient for treatment, his mind has also to be prepared. Explain to the patient in detail about the treatment and also about the after effects of the treatments. This will help the patients cope with the complications in future.

Extraoral examination consists of an inspection of swellings, skin lesions followed by palpation of lymph nodes- submandibular, submental and the cervical chains, TMJ and muscles of mastication.[2] Intra oral examination should be systematic to avoid missing any details. The condition of teeth and soft tissues such as buccal mucosa, tongue, palate and oropharynx should be assessed. The major salivary glands should be examined and the saliva expressed to see the color, consistency, flow etc. Periodontal probing should be done to assess the status of teeth and those with poor prognosis should be indicated for extraction. Carious teeth should be restored and badly decayed once and root tips should be removed. Scaling and root planning should be done and emphasis should be given to maintenance of oral hygiene. Custom made trays can be prepared and patients can be instructed to start using neutral sodium fluoride gels prior to treatment itself.[3]

## TREATMENT PHASE

A patient may report to a dentist just prior to the start of the treatment for dental check-up. In that case, the dentist can take a record of the dental history and examine the patient, as mentioned earlier. The patient should be given scaling and prophylaxis. A thorough debribement of plaque and calculus will reduce the risk of oral infections after therapy. The use of sodium fluoride preparations have to be emphasized. Oral hygiene measures have to be explained to the patients.[4] The teeth with poor periodontal condition, unrestorable carious teeth and root tips have to be extracted. Total extraction in the field of radiation is advisable. Surgical procedures have to be completed 2-3 weeks before the start of chemotherapy or radiotherapy. This will allow 7-14 days of healing period.[5] They should be instructed to discontinue wearing full or partial dentures to reduce trauma to tissues. Patients have to be instructed to take frequent sips of water to reduce the effects of hyposalivation. Study models may be prepared for further use. Dentist and oncologist can discuss about the dental treatments needed to improve the quality of treatment.

**Figure 3:**
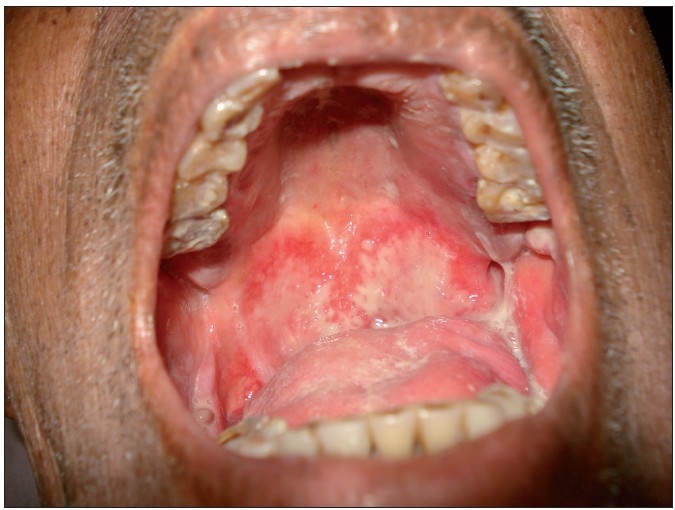
Patient with mucositis and thick pasty saliva

After the start of the treatment, the most important condition that needs the care of a dentist is mucositis or stomatitis.[6] It is a painful and debilitating condition that is a dose and rate limiting toxicity of cancer therapy. Chemo and radiotherapy affects the tissues with high mitotic rate like the oral mucosa.[7] This will lead to atrophy of tissues, ulceration and microbial invasion. Usually mucositis occurs within 5-7 days of start of treatment[8,9] [Figure 3]. The pain in mucositis can be reduced by topical lidocaine preparations. 2% morphine solution has been used topically to reduce pain.[6] Oral cavity must be thoroughly cleaned by a dentist by flushing with povidone iodine preparations and lidocaine. This will help reduce the bacterial colonisation around debri. Soft and liquid diet has to be advised. They are asked to avoid hot and spicy foods, and habits like smoking and alcohol.[10] Patients have to be advised to sip ice cubes. Other preparations used in the treatment of mucositis are water-based lubricants, milk of magnesia, benzydamine, sucralfate suspension etc. The ‘magic mouthwash’ which is a combination of antihistamines, antifungals, topical anesthetics and even antibiotics has been prescribed.[6] Studies have been conducted on biologic response modifiers like granulocyte colony stimulating factors(G-CSF), granulocyte-macrophage colony stimulating factors(GM-CSF) and keratinocyte growth factors (KGF).[7] Amifostine (Ethylol) is thought to act as scavenger for harmful reactive oxygen species that are known to potentiate mucositis.[11]

In patients who receive radiotherapy may develop hyposalivation as radiation results in acinar cell atrophy and necrosis, changes in connective tissue and altered neurologic function. Serous acini are affected earlier resulting in thick viscous secretion. Saliva production is also reduced. Plenty of fluid intake and the use of sugarless gum or candies assist stimulation of residual gland function. Pilocarpine is a parasympathomimetic agent which can be given 5-10 mg tid for increasing salivary secretion. Mouth wetting agents and artificial salivary substitudes can be used. Amifostine has also been studied and is found to reduce salivary gland damage.[11,12] Radiation caries, sequelae of hyposalivation as there is shift toward the cariogenic microorganisms. It can be prevented by using fluoride agents and maintaining oral hygiene.

**Figure 4:**
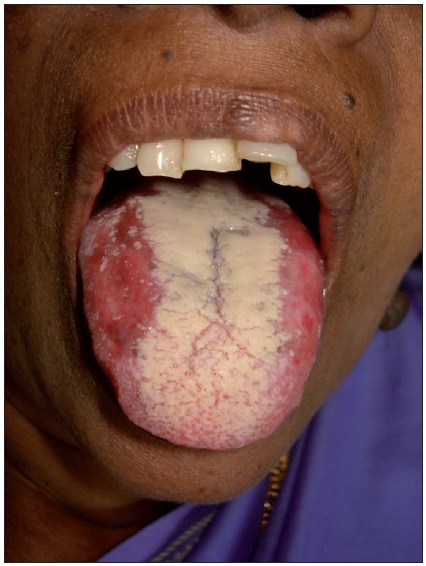
Candidiasis tongue

Candidiasis is the most common oropharyngeal infection in patients receiving radio or chemotherapy.[6,13,14] The predisposing factors for fungal infections are poor oral hygiene xerostomia, immunosupression, poor nutritional status, etc.[13] *Candida albicans* is the most common causative agent. *Candida* infections can manifest as pseudomembranous, erythematous or hyperplastic candidiasis or angular stomatitis [Figure 4]. The patient should be instructed about oral care. The most commonly used topical antifungal is clotrimazole troche, 10 mg, 5 times daily for 14 days. Another topical agent is nystatin(200000-400000IU). Systemic antifungals such as ketoconazole (200-400 mg orally 7-14 days) and fluconazole (50-100 mg 7-14 days) can be given for severe cases. Palliative care givers should take care of the oral conditions of the patient.[15]

## POST TREATMENT

**Figure 5:**
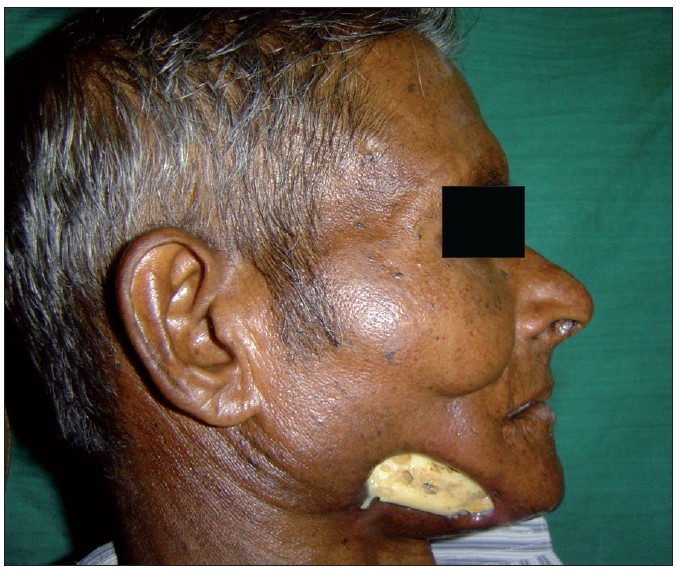
Osteoradionecrosis with exposed mendible

Post radiation osteonecrosis (PRON) is another well known complication of HN RT.[16] RT results in endarteritis that affects vascularity, resulting in hypovascular, hypocellular and hypoxic tissue that is unable to repair or remodel itself. Mandible is most commonly affected due to greater bone density and unilateral vascular supply to each half [Figures 5, 6a and b]. Osteonecrosis and osteomyelitis is also reported in patients receiving bisphosphonate.[17] The risk for PRON is lifelong. Avoidance of trauma, removal of bony sequestrum and use of antibiotics has been advised. In cases associated with pain and progression, hyperbaric oxygen has been advised (20-30 dives at 100% oxygen and 2-2.5 atmosphere of pressure).

**Figure 6:**
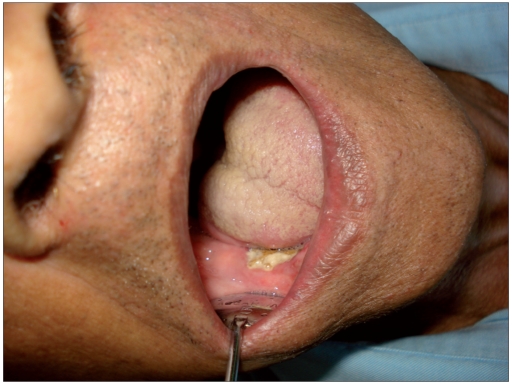
(a) Osteonecrosis mandible

**Figure 6:**
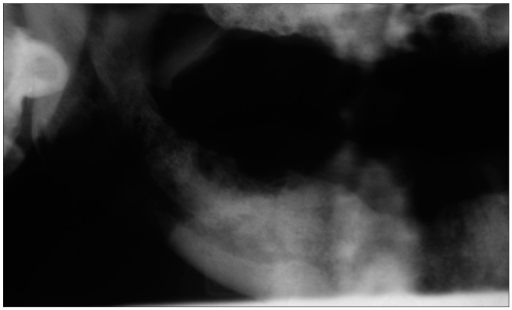
(b) Panoramic radiograph showing osteradionecrosis

**Figure 7:**
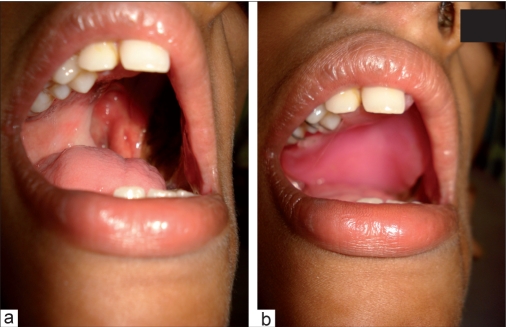
(a) The patient in Figure 1 after maxillectomy of left side; (b) The surgical defect restored with obturator

After the surgical and chemotherapy, the patients may experience difficulties in speaking, swallowing, breathing and esthetics. To a great extent a dentist can help the patient cope with these difficulties. Rehabilitation of the defects occurring after surgery, as far as possible, will help the patient live a better life. Exercises can be taught to the patient with the help of appliances to improve mouth opening. Prosthetic appliances like obturators help to restore the surgical defects in the oral cavity. Surgical defects of other organs like eyes, nose or any other part of the body can be artificially replaced today [Figures 7a and b].

## MANAGEMENT OF PAIN

Pain is the most ‘unpleasant sensory and emotional experience’ as given in the definition by IASP. Relief or reduction in the severity of pain or associated discomforts will be the greatest help that can be offered to a terminally ill patient. A major step toward management of pain is accomplished by good history taking. Listen to the patient for the ‘cause’ of pain. Sometimes the pain may be associated with the disease but sometimes it may be related to the psychological aspects of cancer. We can find out the actual cause only via a good conversation with the patient. If it is psychological then assure the patient. They may have many misconceptions. Clear them all by explaining in simple words. About 90% of the physical pain can be controlled by the application of WHO analgesic ladder.

*Step 1* (Mild pain) – Non-opioids±adjuvant

Example is paracetamol 500 mg-1g 6 hourly. If pain is not controlled in 24 h proceed to step 2.

*Step 2* (Mild to moderate pain) - Weak opioids ± step one medication

A combination of paracetamol, dextropropoxyphene, codeine or tramadol. If step 2 medications are not adequate in 24 h, proceed to step 3.

*Step 3* (Moderate to severe pain) - Strong opioids ± step one medication

Morphine 5 mg four hourly, a maximum daily dose of 30 mg is required.

All medicines should be prescribed after consultation with the patient’s physician.

## CONCLUSION

The palliative doctor gives the ‘touch of God’ as he/she takes care of the patient. In India, palliative care organisations like NNPC and Pallium India are untiringly helping the community. The general dental surgeon does not have enough idea about his part in these treatments. The community is also unaware of the role that a nearby dentist can play. Adequate training programs have to be conducted and awareness has to be created. A trained dental doctor will be a good team mate for the oncologist or radiotherapist or other doctors of the palliative care team.
